# Supplementary material for: Oligomerisation mediated by the D2 domain of DTX3L is critical for DTX3L-PARP9 reading function of mono-ADP-ribosylated androgen receptor
Source: bioRxiv. 2023 Nov 29:2023.11.29.569193. Preprint. [Version 1] doi: 10.1101/2023.11.29.569193 (PMC10705365; doi:10.1101/2023.11.29.569193)
Supplement: Supplement 1 [file media-1.pdf]

Oligomerisation mediated by the D2 domain of DTX3L is critical for DTX3L-PARP9 reading function of mono-ADP-ribosylated androgen receptor

Carlos Vela-Rodríguez[1], Chunsong Yang[2], Heli I. Alanen[1], Rebeka Eki[3], Tarek A. Abbas[3], Mirko M. Maksimainen[1], Tuomo Glumoff[1], Ramona Duman[4], Armin Wagner[4], Bryce M. Paschal[2,*] & Lari Lehtiö[1,*]

[1]Faculty of Biochemistry and Molecular Medicine & Biocenter Oulu, University of Oulu, Finland.

[2]Department of Biochemistry and Molecular Genetics, University of Virginia, USA

[3]Department of Radiation Oncology, University of Virginia, USA

[4]Diamond Light Source, Harwell Science and Innovation Campus, Didcot OX11 0DE, UK.

*Corresponding authors: bmp2h@virginia.edu or lari.lehtio@oulu.fi

Contents

Figure S1. Representative electron density map.

Figure S2. Superimposition of D2 structure with AlphaFold model of DTX3L.

Figure S3. Recombinant protein inputs related to Figure 4.

Table S1. Results from a DALI search.

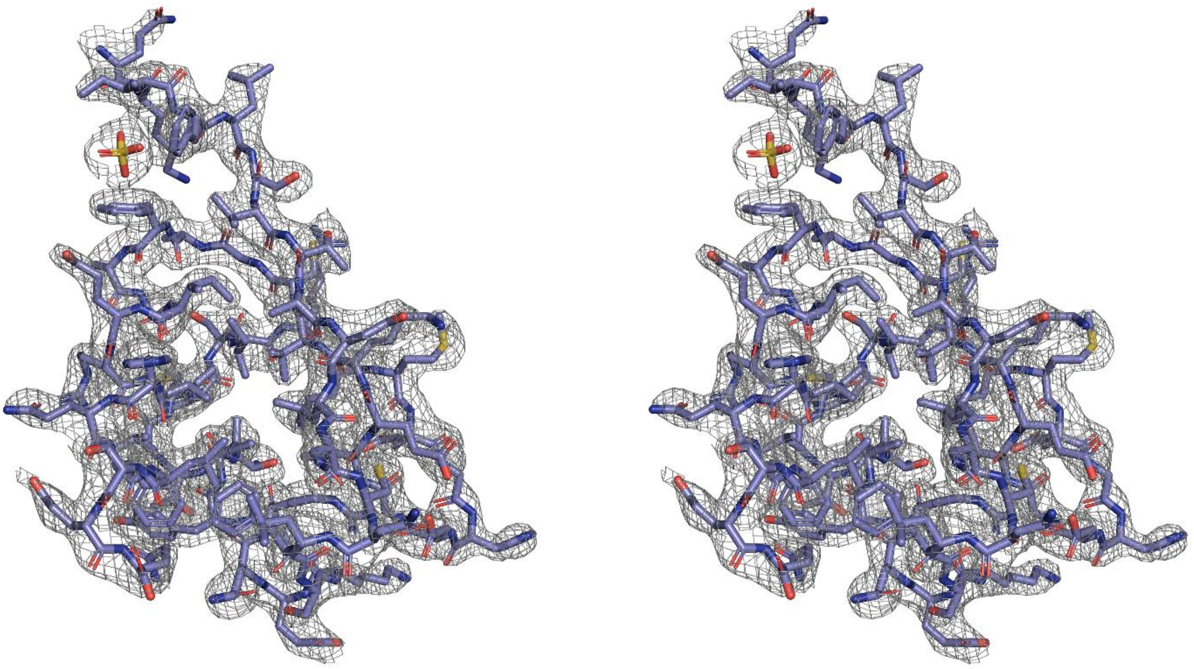

Figure S1. Stereoscopic representation of the electron density map of a monomer of the D2 domain of DTX3L. The electron density map is contoured at 1.0 sigma.

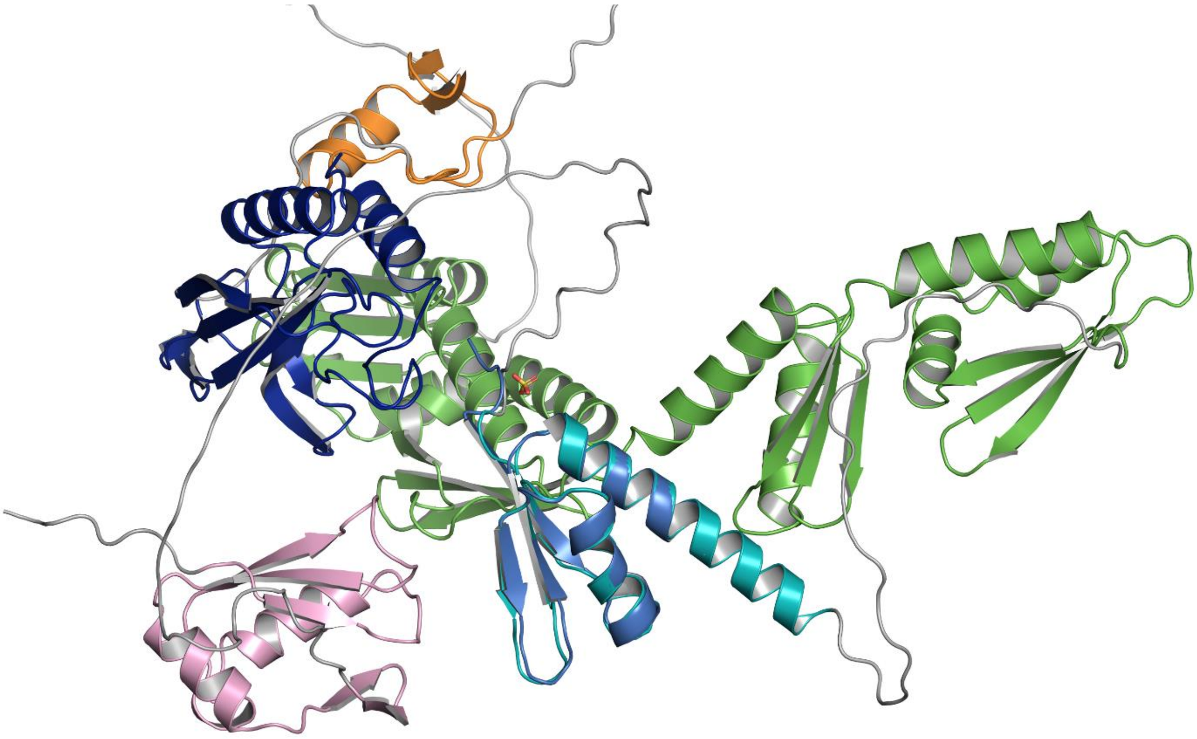

Figure S2. Superimposition of a monomer of the experimentally solved structure (marine) to the predicted full-length structure of DTX3L. Domains of DTX3L are colour-coded as the schematic in **Figure 1A**. AlphaFold2 prediction shows long, flexible regions between the domains of the protein.

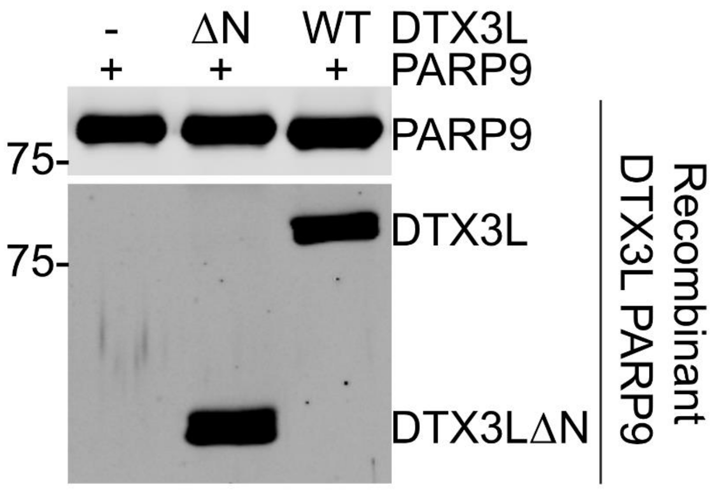

Figure S3. Recombinant protein inputs related to Fig. 4. Recombinant PARP9 alone or combined with DTX3L or DTX3LΔN (10 µM for each protein) was pre-incubated on ice for 6 h, and then diluted to 1 µM each with an extraction buffer [20 mM Tris-HCl (pH 7.5), 100 mM NaCl, 0.5% Triton X-100, 1 mM PMSF, 2 mM DTT, 5 mM EDTA, 5 µg/mL each of aprotinin/leupeptin/pepstatin with veliparib]. Equal volume of protein solution was further diluted with 1x SDS loading buffer and subjected to SDS-PAGE and Western blot analysis.

Table S1. Results obtained from the DALI against the PDB25 dataset.

Chain	Z score	RMSD	Aligned residues	% id	PDB Description
5zdh-A	5.6	3.7	57	2	TYPE II SECRETION SYSTEM PROTEIN D
7eqx-A	5.4	2.7	56	11	CARBOXYPEPTIDASE B
4ec5-A	5.3	3.6	58	14	GENERAL SECRETION PATHWAY PROTEIN D
5hvf-A	5.1	2.6	54	9	CARBOXYPEPTIDASE B2
6w6m-A	5.1	3.0	55	9	TYPE IV PILUS SECRETIN PILQ FAMILY PROTEIN
4av2-A	5.1	2.5	58	9	TYPE IV PILUS BIOGENESIS AND COMPETENCE PROTEIN P
6n10-A	5.0	3.4	57	9	DIPHOSPHOMEVALONATE DECARBOXYLASE MVD1. PEROXISOM
8h6s-A	4.9	3.1	58	9	MALONYL-COA-[ACYL-CARRIER-PROTEIN] TRANSACYLASE
6u45-A	4.9	3.3	55	11	ELONGATION FACTOR 2
6wrv-C	4.9	2.1	50	8	TRANSFERRIN RECEPTOR PROTEIN 1
6wi5-A	4.9	1.9	51	12	DE NOVO DESIGNED PROTEIN FOLDIT4
6wrw-C	4.9	2.2	49	8	TRANSFERRIN RECEPTOR PROTEIN 1
3ce8-A	4.8	2.4	52	8	PUTATIVE PII-LIKE NITROGEN REGULATORY PROTEIN
1jqg-A	4.7	2.6	52	12	CARBOXYPEPTIDASE A
6fij-A	4.7	3.1	54	9	POLYKETIDE SYNTHASE
2n2u-A	4.6	2.1	51	12	OR358
4qbu-A	4.6	3.0	52	17	ZMAA
1i7q-A	4.6	2.6	51	8	ANTHRANILATE SYNTHASE
5oqj-2	4.6	3.5	54	4	DNA-DIRECTED RNA POLYMERASE II SUBUNIT RPB1
1pyt-A	4.6	2.8	55	13	PROCARBOXYPEPTIDASE A
1in0-A	4.5	2.5	50	10	YAJQ PROTEIN
6qum-N	4.4	3.4	56	7	V-TYPE ATP SYNTHASE ALPHA CHAIN
4gx2-B	4.3	2.9	53	9	TRKA DOMAIN PROTEIN
3cj8-A	4.3	2.4	49	16	2.3.4.5-TETRAHYDROPYRIDINE-2.6-DICARBOXYLATE N-
5d4o-A	4.3	2.9	53	4	NITROGEN REGULATORY PROTEIN P-II
7lmx-A	4.3	2.5	55	9	INTEGRIN INHIBITOR
7cyf-D	4.3	2.8	52	8	SLR1512 PROTEIN
2pfd-A	4.3	2.8	55	7	FORMIMIDOYLTRANSFERASE-CYCLODEAMINASE
3f56-B	4.2	2.3	51	4	CSOS1D
5wx8-A	4.2	2.8	56	2	IMMEDIATE-EARLY PROTEIN 2
1wey-A	4.1	3.1	55	4	CALCIPRESSIN 1
1sb6-A	4.1	2.4	50	8	COPPER CHAPERONE SCATX1
3s1e-A	4.1	2.4	52	6	CYTOKININ DEHYDROGENASE 1
2g9o-A	4.1	2.3	50	12	COPPER-TRANSPORTING ATPASE 1
3v2u-C	4.1	2.8	54	11	GALACTOSE/LACTOSE METABOLISM REGULATORY PROTEIN G
7t71-A	4.0	4.3	53	6	MEVALONATE 3.5-BISPHOSPHATE DECARBOXYLASE
8csp-8	4.0	2.9	53	6	28S RIBOSOMAL PROTEIN S34. MITOCHONDRIAL
6fon-A	4.0	2.8	51	8	COPPER CHAPERONE FOR SUPEROXIDE DISMUTASE
6jsh-B	4.0	2.9	55	11	FATTY ACID SYNTHASE SUBUNIT BETA
2l48-A	4.0	3.2	57	5	N-ACETYLMURAMOYL-L-ALANINE AMIDASE
1ec6-A	4.0	2.2	51	12	20-MER RNA HAIRPIN
7np8-B	4.0	3.5	54	15	COENZYME F420-DEPENDENT SULFITE REDUCTASE
4u9r-A	4.0	3.4	55	16	CZCP CATION EFFLUX P1-ATPASE
4zck-A	4.0	2.6	53	17	GTP-BINDING PROTEIN TYPA/BIPA

2wbm-A	4.0	3.0	54	17	RIBOSOME MATURATION PROTEIN SDO1 HOMOLOG
3v97-A	3.9	2.4	48	8	RIBOSOMAL RNA LARGE SUBUNIT METHYLTRANSFERASE L
7uvp-A	3.9	3.4	55	9	TETRACYCLINE RESISTANCE PROTEIN TETQ
8hcn-D	3.9	2.9	55	9	UREASE SUBUNIT GAMMA
2k1r-B	3.9	2.6	49	6	COPPER-TRANSPORTING ATPASE 1
3mah-A	3.9	3.3	53	13	ASPARTOKINASE
8gh6-A	3.9	3.4	53	8	REVERSE TRANSCRIPTASE-LIKE PROTEIN
6eml-p	3.9	3.5	57	9	PRE-18S RIBOSOMAL RNA
7nad-U	3.9	2.5	50	8	25S RRNA
3oq2-B	3.8	2.6	51	6	CRISPR-ASSOCIATED PROTEIN CAS2
7agv-F	3.8	3.5	52	2	K(+)/H(+) ANTIPORTER SUBUNIT KHTT
6cc2-A	3.8	3.4	54	7	CELL DIVISION CONTROL PROTEIN 45 CDC45 PUTATIVE
5wwx-A	3.8	2.9	52	10	RNA-BINDING E3 UBIQUITIN-PROTEIN LIGASE MEX3C
2ywg-A	3.8	3.7	57	14	GTP-BINDING PROTEIN LEPA
5www-A	3.8	2.5	54	11	RNA-BINDING E3 UBIQUITIN-PROTEIN LIGASE MEX3C
6yaq-A	3.8	1.9	49	4	CYTOKININ DEHYDROGENASE 8
5cwa-A	3.8	4.1	58	7	ANTHRANILATE SYNTHASE COMPONENT 1
3kiz-B	3.8	2.5	52	8	PHOSPHORIBOSYLFORMYLGLYCINAMIDINE CYCLO-LIGASE
1eqr-A	3.8	3.4	55	5	ASPARTYL-TRNA SYNTHETASE
5gan-G	3.7	3.0	55	7	SACCHAROMYCES CEREVISIAE STRAIN UOA_M2 CHROMOSOME
4ct8-A	3.7	3.6	57	14	CINA-LIKE PROTEIN
8d8j-F	3.7	2.5	51	14	PROBABLE S-ADENOSYL-L-METHIONINE-DEPENDENT RNA
7ock-L	3.7	2.1	51	6	S-ADENOSYLMETHIONINE SYNTHASE
7qri-A	3.7	2.9	56	9	TRYPTOPHAN 5-HYDROXYLASE 2
1ahu-A	3.7	3.0	55	5	VANILLYL-ALCOHOL OXIDASE
1q5y-D	3.6	2.7	52	8	NICKEL RESPONSIVE REGULATOR
6eld-A	3.6	3.0	52	6	NUCLEOLYSIN TIA-1 ISOFORM P40.U1 SMALL NUCLEAR
6vej-A	3.6	3.5	53	8	PROBABLE RESISTANCE-NODULATION-CELL DIVISION (RND
5yys-A	3.6	3.3	54	7	L-FUCOKINASE. L-FUCOSE-1-P GUANYLYLTRANSFERASE
4yut-A	3.6	3.3	54	6	FAMILY 3 ADENYLATE CYCLASE
6l5d-B	3.6	3.1	52	6	GAS VESICLE PROTEIN
2pff-B	3.6	2.9	54	11	FATTY ACID SYNTHASE SUBUNIT ALPHA
6fht-B	3.5	3.8	55	5	BACTERIOPHYTOCHROME.ADENYLATE CYCLASE
6zvp-A	3.5	2.3	52	4	TYROSINE 3-MONOOXYGENASE
1f93-A	3.5	2.2	53	8	DIMERIZATION COFACTOR OF HEPATOCYTE NUCLEAR
7nhr-A	3.5	3.2	53	15	PUTATIVE TRANSMEMBRANE PROTEIN WZC
6gwj-B	3.5	2.6	52	8	EKC/KEOPS COMPLEX SUBUNIT LAGE3
1v8c-A	3.5	2.9	50	14	MOAD RELATED PROTEIN
1q8l-A	3.5	2.5	52	8	COPPER-TRANSPORTING ATPASE 1
3ui3-B	3.4	2.3	50	8	IMMUNOGLOBULIN G-BINDING PROTEIN G. VIRULENCE-ASS
3c6k-D	3.4	2.2	50	8	SPERMINE SYNTHASE
4qmf-B	3.4	3.5	55	5	KRR1 SMALL SUBUNIT PROCESSOME COMPONENT
6nx5-A	3.4	3.6	51	6	PUMILIO DOMAIN-CONTAINING PROTEIN C56F2.08C
6bwo-A	3.4	2.8	49	8	PYRIDINIUM-3.5-BISTHIOCARBOXYLIC ACID MONONUCLEOT
4pwu-C	3.4	3.0	53	8	MODULATOR PROTEIN MZRA
7qh2-C	3.4	3.2	55	7	LACTATE DEHYDROGENASE (NAD(+).FERREDOXIN) SUBUNIT
6j6g-C	3.4	3.4	55	7	PRE-MRNA-SPLICING FACTOR 8
6s6b-K	3.4	2.4	52	2	CRISPR-ASSOCIATED PROTEIN. CMR5 FAMILY

3afg-B	3.4	3.2	56	13	SUBTILISIN-LIKE SERINE PROTEASE
4lir-B	3.4	3.3	52	6	NUCLEOPORIN NUP53
6teq-A	3.4	3.0	55	7	GALACTOKINASE
2jvz-A	3.4	2.4	52	8	FAR UPSTREAM ELEMENT-BINDING PROTEIN 2
7m7h-B	3.4	3.4	52	10	ERYAI.6-DEOXYERYTHRONOLIDE-B SYNTHASE ERYA3. MODU
4aim-A	3.4	3.9	58	12	POLYRIBONUCLEOTIDE NUCLEOTIDYLTRANSFERASE
1u8s-B	3.4	3.0	53	2	GLYCINE CLEAVAGE SYSTEM TRANSCRIPTIONAL
6pwn-A	3.3	3.9	56	11	SMALL-CONDUCTANCE MECHANOSENSITIVE CHANNEL
3dkx-A	3.3	3.3	55	15	REPLICATION PROTEIN REPB
7wvz-A	3.3	3.5	51	10	BETA-KETOACYL-ACYL-CARRIER-PROTEIN SYNTHASE I
1fd8-A	3.3	2.7	48	8	ATX1 COPPER CHAPERONE
2ko1-A	3.3	2.8	53	8	GTP PYROPHOSPHOKINASE
4zos-A	3.3	3.1	54	2	PROTEIN YE0340 FROM YERSINIA ENTEROCOLITICA SUBSP
6mrj-B	3.3	2.8	53	11	NICKEL-RESPONSIVE REGULATOR
4olp-B	3.3	2.4	49	4	GRPU MICROCOMPARTMENT SHELL PROTEIN
2gx8-C	3.3	2.4	53	4	NIF3-RELATED PROTEIN
7v99-A	3.3	3.3	53	4	TELOMERASE REVERSE TRANSCRIPTASE
6dgd-A	3.3	2.7	53	8	PRIMOSOMAL PROTEIN N'
3wx4-A	3.3	3.5	54	9	ANTI-RESTRICTION ENDONUCLEASE
1yqh-A	3.3	2.5	52	2	IG HYPOTHETICAL 16092
1vr6-A	3.3	3.0	50	4	PHOSPHO-2-DEHYDRO-3-DEOXYHEPTONATE ALDOLASE
3gnw-B	3.3	3.4	51	6	RNA-DIRECTED RNA POLYMERASE
1zav-A	3.2	2.0	50	8	50S RIBOSOMAL PROTEIN L10
2hfs-A	3.2	3.8	53	9	MEVALONATE KINASE. PUTATIVE
4zoq-F	3.2	2.6	47	13	INTRACELLULAR SERINE PROTEASE
7qpr-D	3.2	3.3	49	12	ACT DOMAIN PROTEIN
6s2e-A	3.2	3.7	56	11	DNA POLYMERASE EPSILON CATALYTIC SUBUNIT A
2wbr-A	3.2	3.6	53	8	GW182
6pwj-A	3.2	2.6	54	11	GGDEF AND EAL DOMAIN-CONTAINING PROTEIN
7jtk-i	3.2	2.8	48	6	FLAGELLAR RADIAL SPOKE PROTEIN 1
7cv0-A	3.2	3.8	50	6	TRANSCRIPTIONAL REGULATOR NIAR
2f3j-A	3.2	3.8	50	6	RNA AND EXPORT FACTOR BINDING PROTEIN 2
5u9m-D	3.2	2.4	46	9	SUPEROXIDE DISMUTASE [CU-ZN]
7q4l-A	3.2	5.0	55	7	DEAD END PROTEIN HOMOLOG 1
1kn6-A	3.2	2.9	52	6	PROHORMONE CONVERTASE 1
2hh2-A	3.1	2.5	48	4	KH-TYPE SPLICING REGULATORY PROTEIN
5hb7-A	3.1	3.2	53	8	NUCLEOPORIN NUP53
7nhr-C	3.1	3.5	54	15	PUTATIVE TRANSMEMBRANE PROTEIN WZC
4gzk-A	3.1	2.7	50	12	RNA-DEPENDENT RNA POLYMERASE P2
6wb2-A	3.1	3.3	52	13	HIV-1 VIRAL RNA GENOME FRAGMENT
3opk-C	3.1	3.3	56	4	DIVALENT-CATION TOLERANCE PROTEIN CUTA
6lpn-B	3.1	3.6	54	13	D-2-HYDROXYGLUTARATE DEHYDROGENASE. MITOCHONDRIAL
7m1n-A	3.1	2.9	47	9	PUTATIVE FERREDOXIN
3tvi-D	3.1	4.0	55	15	ASPARTOKINASE
1qfr-A	3.1	2.6	51	8	PHOSPHOCARRIER PROTEIN HPR
5uyy-A	3.1	4.5	54	15	PREPHENATE DEHYDROGENASE
4usj-C	3.1	3.1	53	0	ACETYLGLUTAMATE KINASE. CHLOROPLASTIC
8ba1-A	3.1	3.7	54	6	CLEAVAGE AND POLYADENYLATION SPECIFICITY FACTOR S

8ily-A	3.1	3.8	53	6	SET DOMAIN CONTAINING 1A. HISTONE LYSINE METHYLTR
3j6v-J	3.1	3.0	55	7	28S RIBOSOMAL RNA. MITOCHONDIAL
2raq-B	3.1	2.8	53	9	CONSERVED PROTEIN MTH889
3ihs-A	3.1	3.3	52	8	PHOSPHOCARRIER PROTEIN HPR
1fx2-A	3.1	3.1	55	7	RECEPTOR-TYPE ADENYLATE CYCLASE GRESAG 4.1
7bbb-A	3.1	3.3	50	8	ATP-DEPENDENT RNA HELICASE DBPA
4wd9-A	3.1	2.9	53	11	NISIN BIOSYNTHESIS PROTEIN NISB
2ril-A	3.1	2.6	49	12	ANTIBIOTIC BIOSYNTHESIS MONOOXYGENASE
6me0-C	3.1	4.2	51	14	T.EL4H RNA
2lvw-A	3.1	3.8	55	5	ACETOLACTATE SYNTHASE ISOZYME 1 SMALL SUBUNIT
1yg0-A	3.1	2.3	45	11	COP ASSOCIATED PROTEIN
6cng-A	3.0	2.7	41	10	FATTY ACID KINASE (FAK) B3 PROTEIN
1siz-A	3.0	2.2	44	9	FERREDOXIN
8a8k-A	3.0	3.1	55	4	PAP PHOSPHATASE FROM METHANOTHERMOCOCCUS
6ner-E	3.0	2.8	52	6	BMC-H TANDEM FUSION PROTEIN
4p52-A	3.0	4.1	58	7	HOMOSERINE KINASE
3mcs-B	3.0	3.0	52	4	PUTATIVE MONOOXYGENASE
5anb-K	3.0	3.0	52	10	60S RIBOSOMAL PROTEIN L3
4dnr-A	3.0	2.5	53	9	CATION EFFLUX SYSTEM PROTEIN CUSB
1xpp-D	3.0	3.1	52	13	DNA-DIRECTED RNA POLYMERASE SUBUNIT L
3ced-A	3.0	3.6	55	13	METHIONINE IMPORT ATP-BINDING PROTEIN METN 2
6lxg-A	3.0	2.6	47	2	GTP PYROPHOSPHOKINASE
1fjg-F	3.0	2.5	50	14	16S RIBOSOMAL RNA
6dd5-A	3.0	3.7	53	6	MMB-1 CAS6 FUSED TO MALTOSE BINDING PROTEIN.CRISP
4v1a-k	3.0	2.5	53	4	MITORIBOSOMAL PROTEIN ML37. MRPL37
7r65-A	3.0	3.4	53	6	ADENYLATE/GUANYLATE CYCLASE
6k2e-A	3.0	2.6	50	4	CRISPR/CAS2 PROTEIN
3d45-A	3.0	3.8	53	11	POLY(A)-SPECIFIC RIBONUCLEASE PARN
5hy3-A	3.0	2.0	47	15	MRNA ENDORIBONUCLEASE LSOA
5tl4-A	3.0	2.9	53	4	VANILLATE/3-O-METHYLGALLATE O-DEMETHYLASE
